# Is Infantile Colic an Early Life Expression of Childhood Migraine?

**Published:** 2017

**Authors:** Manijeh TABRIZI, Hamidreza BADELI, Afagh HASSANZADEH RAD, Vahid AMINZADEH, Ali SHOKUHIFARD

**Affiliations:** 1 Pediatrics Growth Disorders Research Center, 17 Shahrivar Hospital, School of Medicine, Guilan University of Medical Sciences, Rasht, Iran

**Keywords:** Colic, Childhood migraine, Child, Relationship

## Abstract

**Objective:**

Migraine is the mosyndrome and infantile colic is a common cause of infantile cry. The pathogenesis of migraine and colic has not been well established and different factors may cause them. There is an association between infantile colic and the occurrence of childhood migraine. We aimed to assess whether infantile colic could be noted as an early life expression of childhood migraine or not.

**Materials & Methods:**

This retrospective case-control study was conducted on 5-15-year-old childrenin Rasht, Iran during 2015-2016. Forty-one cases were children with migraine with or without aura. Overall, 123 Control participants were children with the same age referred to the pediatric clinic for routine care. Data were gathered by a checklist including age, sex, birth weight, family history of migraine, the occurrence of colic and type of feeding during infancy. Data were reported by descriptive statistics and analyzed by Fisher exact test using SPSS ver. 19

**Results:**

Overall, 164 children with the mean age of 8.36± 2.53 yr were enrolled. Seventeen (41.46%) children with migraine vs. 44 (35.7%) children in control group had the positive history of infantile colic and Fisher exact test noted significant relation between migraine and colic. Thirty-three children with infantile colic (46.57%) had the positive family history of migraine, which was significantly higher than 27 children without colic (29.7%). There was a significant relation between infantile feeding and migraine.

**Conclusion:**

There is a probable relation between colic and migraine, therefore, migraine and colic as 2 pain syndromes may have a common pathophysiology and further investigations on this common pathophysiology is justified.

## Introduction

Migraine is the most common childhood recurrent primary headache syndrome. It has polygenic inheritance. It is one of the most common causes of childhood school absenteeism and disabilities. Ten percent of the children aged 5-15-yr-old have migraine (1). A childhood migraine is classified into three groups including migraine with aura, migraine without aura and migraine equivalent syndromes School-aged children usually encounter with migraine without aura (1). Up to now, the pathophysiology of migraine has not been well understood and there is a genetic tendency of highly sensitive neuroglial network and diverse stimulations may cause cortical spreading depression. It suddenly activates a system that initiates the inflammatory process and irritates the peripheral and central afferent pathways. They can cause migraine attack (2).

Infantile colic is a common cause of infantile cry that occurs in 5%-16% of infants. Wessel criteria were defined including cry lasting >3 h per day in 3 d a week for >3 wk during infancy (3). The pathogenesis of colic has not been well-established and different factors such as increased intestinal gas (4), lactose intolerance, (5) and cow’s milk allergy (6) may cause it. Infantile colic can induce parental anxiety noted as a factor for shaking baby syndrome.

According to the limited previous investigations, there is a probable association between infantile colic and the occurrence of childhood migraine, which needs verification. Therefore, we aimed to assess whether infantile colic could be noted as an early life expression of childhood migraine or not.

## Materials & Methods

This retrospective case-control study was conducted on 5-15-yr-old children referred to Neurology Clinic of 17-Shahrivar Hospital, Rasht, Iran during 2015-2016.

Cases were children with migraine with or without aura. Migraine was indicated by the International Classification of Headache Disorders II (ICHD II) criteria and the diagnosis of pediatric neurologist. The ICHD II criteria include 5 migraine attack which lasts for 4-72 h. Patients should have at least 2 following characteristics including unilaterality, ponding, deterioration of physical activity, and moderate to severe headache. In addition the existence of one of the following symptoms including nausea or vomiting, photophobia, phono phobia is necessary. Also, the migraine induced headache should not be related to the other causes of headache. In addition, the existence of one of the following symptoms including nausea or vomiting, photophobia, phonophobia is necessary. In addition, the migraine-induced headache should not be related to the other causes of headache. Patients with the migraine included in this study should not have had any concomitant acute or chronic disease. Patients with secondary pure tension headache were excluded.

Control group included children with the same age referred to the Pediatric Clinic for routine care. Inclusion criteria were noted as no history of recurrent headache or other acute or chronic diseases. Controls with secondary pure tension headache were excluded. Data were gathered using a checklist including age, sex, birth weight, family history of migraine, the occurrence of colic and type of feeding during infancy.

The history of infantile colic was noted by Wessel criteria and vivid family report. Wessel criteria defined colic as>3 h per day cry in 3 d a week for >3 wk during infancy(3).

 The history of familial migraine was noted based on ICHD II criteria for migraine with or without aura. Ethical approval was obtained from Guilan University of Medical Sciences (93113007, 18 Feb 2015) and consent letter was obtained from each participant or related parents. Data were reported using descriptive statistics (number, percent, mean, standard deviation) and analyzed by Fisher exact test using SPSS v 19 (Chicago, IL, USA).P-value< 0.05 indicated statistical significance and 95%confidence interval was noted.

## Results

Overall, 164 children aged 5-15-yr-old with the mean age of 8.36± 2.53 yr were enrolled. Most of the participants (90) were girl (54.88%). The mean birth weight of the participants was 3162±0.484 gr. The case group consisted of 41 children with migraine including 19 (46.34%) girls and 22 (53.64%) boys. Control group consisted of 123 healthy children including 71 girls (57.72%) and 52 boys (42.28%).

Seventeen (41.46%) children with migraine vs. 44(35.7%) children in control group had the positive history of infantile colic and Fisher exact test noted significant relation between migraine and colic (P=0.049). Thirty-four children with infantile colic (46.57%) had the positive family history of migraine that was significantly higher than 27 children without colic (29.7%) (P=0.001). There was a significant relation between type of feeding during infancy and migraine(P=0.001) Most of the healthy children without migraine were breastfed and the frequency of breastfeeding in children with migraine was the least (14.63% vs. 39.02%, respectively) (Table 1).

**Table 1:** Demographic characterizes of participants

Variables	Groups		Total n (%)	P-value*
	Children with migraine n (%)	Healthy children N (%)		
History of Infantile colic				
Yes No total	17(41.46) 24(58.54) 41(100)	44(35.7) 79(64.3) 123(100)	61(37.2) 103(62.8) 164(100)	0.049
Sex				
Girl Boy Total	19(46.34) 22(53.64) 41(100)	71(57.72) 52(42.28) 123(100)	90(54.87) 74(45.13) 164(100)	0.211
Type of feeding				
Breastfeeding Formula feeding Both	6(14.63) 16(39.02) 19(46.35) 41(100)	108(94.8) 15(5.2) 0(0) 123(100)	114(69.5) 31(18.9) 19(11.6) 164(100)	0.001
Family history of migraine				
Yes No Total	39(95.12) 2(4.88) 41(100)	34(27.65) 89(72.35) 123(100)	73(44.51) 91(45.49) 164(100)	0.001

* fisher exact test

## Discussion

Infantile colic is the leading cause of pain and cry in healthy infants and may be considered as a pain syndrome (7). Migraine is the leading primary childhood headache that induces daily activity dysfunctions such as school absenteeism. Although the relation between the occurrence of these two pain syndromes (migraine and colic) was noted by previous investigations, there is no definite consensus on this relation.

In our study, 41.6% of children with migraine versus35.7% of patients without migraine had infantile colic. Children with migraine had significantly higher rate of infantile colic comparing healthy children. These results were consistent with previous investigations (7-11). Romanello et al (2013) which assessed overall, 208children aged 6-18-yr-old were assessed and noted children with migraine had higher rate of infantile colic (72.6%) comparing with healthy children (26.5%).

Results showed no significant relation between colic and tension type headache (7). Furthermore, Jan et al (2001) noted that 52% of children with colic and 20% of healthy ones had infantile migraine and significant relation was noted (8).

Bruni et al mentioned that the the history of infantile colic in children with migraine was higher than tension type headache and the positive history of infantile colic was significantly higher in children with migraine (38%) comparing normal children (26.9%) (9).

In a prospective study by Guiddetti et al, 53% of hyperreactive children (spontaneous repeated cry) comparing 11% of healthy children had migraine. In addition, the frequency of childhood periodic syndrome, which may be a pre-migraine condition, was higher in hyperreactive infants (12).

Regarding the scientific hypothesis, although infants with colic may have an experience of the same sensitization in the perivascular nerve terminals in the gut instead of CNS, it seems it should be well-investigated (7). Because the known molecules intervening the justification of sensory activities such as Calcitonin

Gene-related Peptides (CGRP) could potentially affect the pathogenesis of pain. CGRP may be released during the migraine attacks and CGRP antagonists are the pain controllers (13). It also potentially may be involved in the pathogenesis of abdominal pain regarding the induction of neurogenic sensory nerve inflammation in the gut (14).

In addition, in our study, family history of migrainein children with migraine was significantly higher. It may potentially indicate the genetic origin of migraine. Moreover, 46.57% of children with infantile colic had the positive family history of migraine that was higher than children without colic (29.7%). In a case-control study, the vivid parental history report of infantile colic (the same with our policy), 29 children with migraine and 29 healthy children were assessed. Results showed higher frequency of family history of migraine in children with colic comparing the normal children (41% vs. 31%, respectively P=0.03) (8).

Gelfand et al (2012) compared children with and without colic were compared. The rate of maternal migraine in children with colic was 2.6 times higher than that of children with no history of colic. The maternal migraine associated with infantile colic. According to the genetic tendency of migraine, colic may be an early age presentation of migraine (15).

In this study, there was a significant relation between infantile feeding and migraine. The frequency of breastfeeding was significantly lower in children with migraine that was similar with previous study. Romanello et al noted higher frequency of formulawas noted feeding in children with migraine. They considered breastfeeding as a potential early prohibiting factor of childhood migraine (7).

 The treatment of infantile colic includes drug therapy, nutritional modifications, and behavioral strategies(16). Scientific documents mentioned the effectiveness of casein hydrolysate formula for treating infantile colic regarding the effect of casein on the allergy induced by cow’s milk allergy in infants (17). Feeding with cow’s milk based formulas could relatively justify our findings that noted the higher rate of migraine in formula fed children. The other probability might be because of the protective effect of the ingredients of breastfeeding confirmed. (7)

Triptans, which are 5-HT1B agonists, can be effective in an acute and abdominal migraine (18). Besides, in a report, an infant with colic responded to antimigraine therapy (cyproheptadine) (19). Before selecting antimigraine therapy are mandatory for infantile colic. The limitations of this study are as follows: Although, investigators believe that a prospective cohort study may indicate more concise results, according to the higher sample size in this retrospective case-control study comparing the previous investigations, these results are valuable. In addition, the definite diagnosis of infantile colic was mentioned by the vivid parental reports that might be more concise if investigators assessed infants longitudinally; therefore, investigators excluded the suspicious cases.

**In conclusion**, it seems colic and migraine as two pain syndromes have a common pathophysiology and further investigations on this common pathophysiology can berecommended
